# Employment and deployment of additional staff roles in general practice: a realist evaluation of what works for whom, how, and why

**DOI:** 10.3399/BJGP.2024.0562

**Published:** 2025-01-28

**Authors:** Imelda McDermott, Sharon Spooner, Kath Checkland

**Affiliations:** Centre for Primary Care and Health Services Research, University of Manchester, Manchester.; Centre for Primary Care and Health Services Research, University of Manchester, Manchester.; Centre for Primary Care and Health Services Research, University of Manchester, Manchester.

**Keywords:** additional roles, general practice, new roles, primary health care, qualitative research, workforce

## Abstract

**Background:**

The Additional Roles Reimbursement Scheme (ARRS) was introduced in England in 2019 to alleviate workforce pressures in general practice by funding additional staff such as clinical pharmacists, paramedics, first-contact physiotherapists, and from 1 October 2024 the scheme funds recently qualified GPs. However, the employment and deployment models of ARRS staff present ongoing complexities and challenges that require further exploration.

**Aim:**

To explore the decision-making processes behind primary care networks (PCNs) and general practice staffing choices, and how these choices influence the operationalisation of ARRS.

**Design and setting:**

This was a qualitative case study across four PCNs in England using a realist evaluation framework.

**Method:**

Data collection took place between September 2022 and November 2023. Semi-structured interviews were conducted with PCN clinical directors, GPs, practice managers, and ARRS staff (*n* = 42). Transcripts were analysed using a realist evaluation framework to identify the context–mechanism–outcome configurations.

**Results:**

Direct employment models fostered staff development and retention, contingent on established trust among practices. Subcontracting was favoured to mitigate employment risks but could lead to unintended consequences such as conflicting accountabilities and less integration with existing GP practice staff. The optimal deployment model involved rotations across a limited number of GP practices, ideally two, with one serving as a base, ensuring consistency in training and management.

**Conclusion:**

This study provides novel insights into the complexities of different employment and deployment models of ARRS staff. These findings will be invaluable for creating a sustainable GP practice workforce and informing future workforce strategies as the scheme expands to include recently qualified GPs.

## Introduction

Increasing pressures on the workforce in general practice in England are being addressed, in part, by extending the use of practitioners who are not GPs. The Additional Roles Reimbursement Scheme (ARRS) was introduced in England in 2019 to fund additional staff in general practice, initially focusing on five ‘new’ roles and it has been expanded to 17 additional roles.^1^ These roles included clinical pharmacists, physician associates, first-contact physiotherapists, paramedics, and social prescribers. From 1 October 2024, additional funding of £82 million is available to support the inclusion of recently qualified GPs in the ARRS.^2^

This funding is available through a separate Network Contract Directed Enhanced Service, on top of the standard General Medical Services contract, to cover a wide range of initiatives designed to enhance and expand primary care services delivered through primary care networks (PCNs). PCNs are groups of GP practices that work together to deliver integrated community-based health care for populations of 30 000–50 000 people. However, PCNs cannot directly receive ARRS funding as they are non-statutory organisations and hence do not have a formal legal status unless they choose to establish themselves as a legal entity, such as a limited liability partnership or a social enterprise. Hence, although funding can only be accessed via PCNs, PCNs cannot directly employ ARRS staff. Instead, the employment of ARRS staff must be facilitated through alternative arrangements, such as through member GP practices or other legal entities within the PCNs, such as GP federations, that is, groups of GPs who have established a formal collaboration such as a community interest company or limited liability partnership.

**Table:** How this fits in

Previous research has identified variations in how Additional Roles Reimbursement Scheme (ARRS) staff are employed and deployed. This study adds to existing knowledge by exploring the decision-making processes behind PCN and GP practices staffing choices. Our study found that decisions about ARRS employment and deployment models are driven by factors such as greater influence over staff development and previous experience with ARRS roles. PCNs and GP practices need to consider how best to support and integrate recently qualified GPs in ARRS roles to ensure adequate support for career progression and long-term retention in general practice.

Our study builds on existing studies that have explored the employment models of ARRS staff by Bramwell *et al* (2023)^3^ and Baird *et al* (2022).^4^ Box 1 describes these different models and their advantages and disadvantages. The employment of ARRS staff varies widely but primarily follows two models: direct employment or subcontracting.^3^^,^^4^ Direct employment (for example, via federations or GP practices) was found to facilitate a more integrated approach, where ARRS staff are embedded within the practice team and considered part of the core workforce. However, both studies indicated a preference for a model that limits the extent to which individual GP practices bear risks and responsibilities.^3^^,^^4^ Baird *et al* found that there can be significant challenges in terms of managing day-to-day human resource responsibilities and ensuring consistent terms and conditions across different roles^4^ when directly employed ARRS staff are deployed across more than one GP practice.

**Box 1.** Employment models of ARRS staff according to Bramwell *et al*^3^ and Baird *et al*^4^

**Employment model**	**Description**	**Advantages**	**Disadvantages**
**Direct employment**	ARRS staff are directly employed by a lead GP practice or a formally constituted GP federation (groups of GPs who have established a formal collaboration such as a community interest company or limited liability partnership)	Simpler HR management as staff are employed by a single entity	Requires the GP federation to be a formal legal entity As ARRS staff are deployed across >1 GP practice, there can be challenges in managing day-to-day HR responsibilities such as annual leave
**Subcontracting**	ARRS services are contracted out to external organisations such as health trusts or third-sector organisations	Limits employment risks for individual GP practices	May lead to less integration and how ARRS staff can be utilised to meet local needs

*ARRS = Additional Roles Reimbursement Scheme. HR = human resources.*

In the early days of implementation, subcontracting new staff from another organisation was considered advantageous due to uncertainties and unfamiliarity with the terms of employment for ARRS staff. This approach allowed PCNs to mitigate risk during the early stages of implementation.^3^ Subcontracting models, although potentially offering greater flexibility and access to specialised skills, can create issues related to variation in terms and conditions, particularly when staff are sourced from NHS organisations not subject to Agenda for Change, which can lead to tension and inequity among staff, having an impact on team cohesion and morale.^4^ Moreover, some PCNs did not have the skills to manage a contracting process.^3^ Centralised employment models, where staff are employed by, for example, GP federations, can mitigate some of the challenges, such as competition between practices offering different salary levels. However, it could also create a sense of distance between ARRS staff and the primary care teams they work alongside, potentially hindering effective collaboration and integration. In areas where there are shortages of particular roles, sharing staff at scale or across a geographical area where staff rotate through primary and secondary care at agreed intervals could prevent the workforce from being depleted and reduce the administrative burden of recruitment, human resources, and management.^5^

Having employed staff in particular ways, PCNs must also decide how they are deployed. Staff directly employed by a single lead practice might spend some time working in each of the PCN member practices, or each staff member might work across a small number of practices. Alternatively, a central model might be adopted for some types of staff in which patients from other practices attended the lead practice for a particular service. Where staff are contracted from an external organisation, the contract would need to specify how rotation between practices was to be managed and the extent to which named staff would be provided in a consistent way. Some contracted staff might move between primary and secondary or community care services, spending only a limited amount of their time in the general practice setting. An example would be paramedics employed by ambulance trusts but splitting their time between primary care and their existing roles in emergency services. This type of rotational model was seen as beneficial for skill development as the diverse range of patients and tasks encountered can enhance paramedics’ clinical skills, autonomy, and staff retention as it was thought to improve job satisfaction and retention within both general practice and ambulance services.^6^ Some contracted services, such as social prescribing, might be provided from a centralised building such as a community services clinic or a building belonging to a community organisation.^7^

The variability in ARRS employment and deployment models creates a complex landscape where understanding the factors driving successful outcomes is challenging. There is unlikely to be ‘one best way’ to operationalise the ARRS. Moreover, what works well from one perspective (for example, that of the PCN) might be less advantageous from the perspective of an individual practice or the ARRS staff. This article addresses this critical gap by offering an in-depth exploration of the decision-making processes behind PCN and general practice staffing choices. By shedding light on how these decisions influence the operationalisation of ARRS, this research contributes valuable insights to optimise workforce strategies and enable a more effective and tailored approach to employing ARRS staff in general practice.

## Method

We used realist evaluation as a framework to capture and explore the variations in the employment and deployment models of ARRS staff. A realist approach to evaluation seeks to explore ‘What works, for whom, in what respects, to what extent, in what contexts, and how?’^8^ and therefore offers a language with which to explore the operationalisation of ARRS in practice. A fundamental building block of a realist account is a programme theory or collection of theories that may form a basis on which to build understanding or form plausible explanations of the contexts and mechanisms that lead to particular outcomes.^9^

## Data collection

We adopted a pragmatic approach in our data collection by sending an expression of interest to the ORCHID network, a database which contains information on roughly 1858 UK general practices. The same database was used in the quantitative analysis as part of the wider project that looks at health outcomes and cost–benefit analysis.^10^ We screened responders based on criteria such as practice size, population, PCN characteristics, ARRS staff types, employment models, and success in utilising ARRS funds.

We chose four PCNs to study in depth. This allowed us to understand the contexts within which the decisions discussed in the interviews had been made. The interviews used a realist approach.^11^ The focus of the interviews was on:

- why PCNs make the decision to employ a particular ARRS staff;
- the perceived advantages/disadvantages of different employment, deployment, and management models for ARRS staff (for PCNs, practices, and the staff themselves); and
- perceptions of the likely outcomes associated with approaches.

We interviewed three groups of participants. We started by interviewing PCN clinical directors to develop initial programme theories. Subsequent interviews with GPs and practice managers refined the initial programme theories. Finally, our interviews with ARRS staff elicited programme-inhibiting factors and unintended consequences.

A total of 42 interviews were conducted across four PCNs. Data collection was conducted by the first author and took place between September 2022 to November 2023. Audio-recorded interviews (with consent) were transcribed verbatim by the university-approved transcription company. Transcription texts were thoroughly checked and anonymised by a researcher (the first author).

The site characteristics were as follows:

- site 1: a GP federation in the process of becoming a community interest company and that acted as the main employer for most ARRS staff in the PCN;
- site 2: a PCN in the process of becoming a limited company and each individual GP practice within the network was responsible for employing one type of ARRS staff;
- site 3: each GP practice in the PCN independently employed its own ARRS staff; and
- site 4: a GP federation that directly employed its ARRS staff.

Table 1 summarises the list of participants we interviewed.

**Table 1.** Interview participants

**Job role/title**	**Site 1**	**Site 2**	**Site 3**	**Site 4**	**Total by job role/title**
**PCN clinical director**	1	2	1	0	4
**GP partner**	0	1	1	0	2
**Practice/business manager**	2	2	1	1	6
**Nurse**	1	0	0	1	2
**Nurse associate**	0	0	1	0	1
**Physician associate**	0	0	0	1	1
**Clinical pharmacist**	1	3	1	0	5
**Pharmacy technician**	0	2	1	0	3
**First-contact physiotherapist**	1	2	1	0	4
**Paramedic**	0	0	0	1	1
**Social prescribing link worker**	0	2	1	1	4
**Health and wellbeing coach**	1	2	1	0	4
**Care coordinator**	0	3	1	1	5
**Total by sites**	7	19	10	6	42

*PCN = primary care network.*

## Data analysis

Anonymised interview transcripts were imported into qualitative data analysis programme NVivo 12. Interview transcripts were analysed by the first author using a realist evaluation framework to identify the context–mechanism–outcome (CMO) configurations. The first author read the transcripts and coded interview sections as potential contexts, mechanisms, and outcomes within each case study site and by practitioner type. CMO configurations were developed, and commonalities were identified and grouped together. These initial CMO configurations were shared with the second author for discussion and refinement at regular team meetings. Findings are presented here as context (C), mechanism (reasoning or resources) (M-reasoning or M-resources), and outcomes expected (O).

## Results

### Employment models

Direct employment model of ARRS staff by a GP federation or a lead GP practice (C) facilitates staff development (M-reasoning), leading to improved staff retention (O):

*‘For me personally, I think you get a lot of added value when you invest in people directly and they’re part of the team and you know their name and you can train them and you can go and ask them for advice … You don’t get that with an outsourced model. And that continuity and those relationships are invaluable, I much prefer, even though it’s harder work, I much prefer having individuals employed in-house and part of the team.’*

(PCN clinical director, site 1, identification [ID]1)

In the context of direct ARRS staff employment by a lead GP member practice (C), the decision on which practice should be employing which ARRS staff (O) is dependent on whether the practice had experience employing the type of ARRS staff and hence knows what to expect (M-reasoning):

*‘It was purely that the practice manager said, “oh well we’ve got good contracts quite easy at our practice, let’s do that” … It wasn’t a conscious choice particularly, and there just does tend to be quite a good spirit of … I’ll do this, we’ll do this, working together.’*

(PCN clinical director, site 3, ID32)

When a single organisation is employing a type of ARRS staff (C), the staff can be employed under the same terms and conditions, and, hence, issues such as training and line management can be more well coordinated (O). This approach relies on mutual trust among practices, built on a history of working together in the past and practices sharing the same values and ethos (M-reasoning):

*‘Staff under the same contract can do more things like training for other staff. It seemed like the best way to do it. We thought if you wanted to make the pharmacist, for example, a team it would be better that they were employed under the same conditions by the same people, with the same managers … And so it’s better that they’re employed by one practice … but that then comes back to the practices trusting each other and trusting that they will treat people reasonably and that it works OK. But it does seem to work better like that … Practices having the same ethos and have worked together in the past — more straightforward decision making because there’s trust.’*

(PCN clinical director, site 2, ID10)

A subcontracting model was typically used to employ first-contact physiotherapists, paramedics, and social prescribers. This model was chosen because practices within the PCNs did not have experience in employing and recruiting this type of ARRS staff; hence, they had unclear expectations of what the staff could deliver and also what clinical supervision or training needed to be provided for the staff (M-reasoning). In the context of paramedics (C), subcontracting was chosen as PCNs did not want to destabilise existing ambulance services (M-reasoning):

*‘I would like to see a home visiting service with paramedics … but we’d have to employ paramedics and there’s quite a challenge at the moment without destabilising local ambulance services.’*

(Practice manager, site 2, ID13)

The reasoning was more complex for social prescribers. Subcontracting and not having social prescribers based at the GP practice allowed patients to be seen in the community, potentially improving engagement (M-reasoning). However, direct employment of social prescribers, as with other ARRS staff, provides greater control over the service and allows patient records to be integrated into the existing GP system, facilitating feedback for clinicians (O). The trade-off for PCNs with direct employment of social prescribers was the need for PCNs to establish and develop necessary resources from scratch (M-resources):

*‘That’s what social prescribing is … it’s about holistic wellbeing … it’s promoting that wellbeing is not all medical, it’s about more holistic care, and there’s more to that than kind of being reliant on coming to the GP when there’s a problem.’*

(PCN clinical director, site 3, ID32)

### Deployment models

ARRS staff in our case study sites generally rotated across two to three different practices. However, when rotations occur across practices with diverse patient populations (C), ARRS staff would find that different practices had different expectations, requiring them to perform their role differently in each practice (O):

*‘The other difficulty has been through the years to keep my job fairly standardised because the practices, some of them have very different populations. The practice where I am phoning you from today* [has a] *very young population, lots of young families, et cetera, whereas in* [Practice A]*, for example, or in* [Practice B]*, there’s elderly patients, lots of care homes, et cetera. So of course, they have different needs from me.’*

(Clinical pharmacist, site 3, ID35)

It is worth noting the type of rotational model often seen in paramedics, whereby they not only rotate across various GP practices but also alternate between GP work and ambulance service, was not something that was present in our case study sites.^12^

An alternative deployment model involved ARRS staff being primarily based at one regular base site, which would provide support for the training while also rotating to a second practice every 6–9 months (C) to learn the different ways of working and build contingency (M-reasoning). This approach would ensure that practices had familiarity with the ARRS staff in case they needed to step in and support them, enable learning and/or sharing best practices learnt elsewhere, and provide continuity for practices, patients, and the ARRS staff themselves (O). For this to work, it requires having a team responsible for workforce planning (M-resources):

*‘The patients, obviously we are seeing patients and doing the reviews with them. A lot of those reviews do require follow-up, so it’s beneficial to be able to have that follow-up with the same pharmacist; we know the background, and they don’t have to go over it all again. In terms of the practices, the practice gets used to us as pharmacists and we all have different backgrounds … having that one pharmacist in or just two pharmacists it means the practice then knows who to refer certain patients to, if they want somebody to speak to someone about pain relief versus respiratory, somebody who is a prescriber or not a prescriber for example, it means the practice is used to who they’re working with. And then for us as pharmacists just having two places of working and two ways of working is much better and much easier.’*

(Clinical pharmacist, site 1, ID6)

When ARRS staff were deployed across more than two practices whereby an ARRS staff member works only 1 day a week in a GP practice (C), they had to ensure that they were giving the hours needed by the practice (O); otherwise, the practice can feel that they were not getting the ARRS staff time needed for their patients (M-reasoning):

*‘I’m trying to make sure that every practice gets their exact proportion of my time that they are allocated to do, which is a bit tricky sometimes … And obviously the practice where I work on Mondays would otherwise lose out because every time there’s a Bank Holiday they will lose out and I try to keep a balance of my hours so every time a practice is one working day up or down then I sort my days that week and balance the hours again.’*

(Clinical pharmacist, site 3, ID35)

Deploying ARRS staff across a large number of GP practices (C) can trigger a sense of being overwhelmed and thinly spread among ARRS staff (M-reasoning). This could lead to compromised care quality, heightened clinical risks, and a perceived lack of integration with existing GP practice staff, and in extreme cases, this can escalate to service suspension owing to safety concerns (O):

*‘And the challenge we have is … I’m in practice one out of ten, so I’m physically sat in one, to ensure every patient in the network gets a slice of the cake so to speak, they insist we can book anyone in anywhere. So I could be sat in practice one, but have patients from every other practice, so my ability to discuss patient cases or get some clinical support should I need it* [is limited because] *I can’t go to the GPs that are in the building because it’s not their patient. So it’s a very siloed way of working to be honest … it actually got to the point yesterday where as a clinical lead, me and my colleague are both clinical leads for the service and we’ve said we want to suspend the service, because it’s now not safe clinically.’*

(First-contact physiotherapist, site 2, ID26)

Deploying ARRS staff across multiple GP practices can inadvertently lead to challenges such as conflicting priorities and a sense of isolation among ARRS staff. To address these issues, site 2 had started procuring an estate to co-locate ARRS staff in a central hub to foster team cohesion and reduce feelings of isolation. However, there must be a balance between centralised co-location and time spent at individual practices:

*‘We’ve just recently procured an estate for all of our ARRS staff … we’ll be able to have some of the ARRS teams working together, which I think will resolve a lot of these problems … because we’ve had some feedback that there’s a bit of isolation for some of the ARRS staff … Whereas if they get to work … in an estate … they’ll feel much more a part of a team and less isolated. So that was the thought behind co-locating but some practices don’t want all of their ARRS staff there all of the time. So we’ve had to come up with a bit of a compromise … a bit of a sixty/forty split* [staff based at the new estate for 60% of their time] *that they’ll be based at the new estate and* 40% *based in practice. And that’s taken a bit of negotiation again because some practices have said, no, we want them here all the time, some practices have said, we’ve got no space, take them all, take them* 100%*.’*

(Practice manager, site 2, ID13)

## Discussion

### Summary

Our study found that direct employment models provide greater influence over staff development and retention, potentially enhancing continuity of care. However, their success relies heavily on established trust among practices within the PCN or federation established through a history of working together. In contrast, subcontracting was chosen to prevent disruption of existing services or when there was a lack of experience in employing specific ARRS staff and hence unclear expectations of what it involved. However, subcontracting could lead to unintended consequences such as conflicting accountability and overlooked training needs. The most common deployment model of ARRS staff is a rotational model across two to three GP practices. This model could provide continuity for patients and staff, enable learning and sharing of best practices, and build contingency plans. Rotating across more than two GP practices with diverse populations can make it challenging to maintain standardised job responsibilities. One way to address this is by having the staff based in one practice as their base, which can provide improved stability for human resources, admin management, training, etc. Building confidence in the capabilities of ARRS staff is important for both GPs and patients. Ensuring adequate training and support for these roles is paramount. This requires allocating dedicated time in everyone’s schedule — time for supervising, teaching, and offering support, as well as time for receiving supervision, teaching, and support.

### Strengths and limitations

The strength of the study is in the use of realist methodology. This allows us to go beyond simply describing employment and deployment models, and to explore the who, how, and why questions, which have not been explored before. This study focused on four PCNs, which, although they had different contextual characteristics, may not reflect the full range of employment and deployment practices that apply across all PCNs and for all the different types of ARRS staff. Moreover, as only one researcher analysed all the transcripts, it could limit the development of CMO configurations.

### Comparison with existing literature

Earlier studies found that reasons for choosing particular employment models varied; however, the preference was for approaches that limited employment risks to individual GP practices but mostly was a pragmatic decision based on local circumstances.^3^^,^^4^ On the other hand, our study shows that, as ARRS develops, the desire for greater influence over staff development and retention often drives the choice of employment models. Prior experience with specific ARRS staff also plays a significant role in decision making. Additionally, we found that rotating staff across a limited number of practices, ideally two, with one serving as a base, helps ensure consistency in training, administrative, and management processes. Wider issues identified in our study, such as lack of integration with existing GP practice staff and lack of career progression, support, and supervision for ARRS staff, are similar to those found by Jones *et al.*^13^

### Implications for practice

Understanding the intricacies of ARRS staff employment and deployment models is fundamental for building a sustainable and effective general practice workforce, improving patient care, and ensuring the retention of ARRS staff. Many ARRS-funded roles are relatively or entirely new in UK general practice and appointed practitioners have frequently not undertaken significant training or had experience of working in general practice, and some may be newly qualified. PCNs and GP practices need to facilitate the integration and embeddedness of ARRS roles into general practice and develop their skills and knowledge to ensure safe and effective patient care. ARRS staff need to feel valued and supported to ensure their retention and job satisfaction. This includes providing induction, mentoring, ongoing support and supervision, and access to professional training and appraisal. The inclusion of recently qualified GPs in the ARRS funding from October 2024 presents additional challenges. PCNs and GP practices need to determine how recently qualified GPs can be adequately supported and mentored within their ARRS roles to enable them to build long-term careers in general practice.
